# Novel Gas Sensor Arrays Based on High-Q SAM-Modified Piezotransduced Single-Crystal Silicon Bulk Acoustic Resonators

**DOI:** 10.3390/s17071507

**Published:** 2017-06-26

**Authors:** Yuan Zhao, Qingrui Yang, Ye Chang, Wei Pang, Hao Zhang, Xuexin Duan

**Affiliations:** State Key Laboratory of Precision Measuring Technology & Instruments, Tianjin University, Tianjin 300072, China; zhaoyuan@tju.edu.cn (Y.Z.); yangqingrui@tju.edu.cn (Q.Y.); cy0803@tju.edu.cn (Y.C.); weipang@tju.edu.cn (W.P.); haozhang@tju.edu.cn (H.Z.)

**Keywords:** e-nose system, piezotransduced single-crystal silicon bulk acoustic resonators, gas sensing, MEMS

## Abstract

This paper demonstrates a novel micro-size (120 μm × 200 μm) piezoelectric gas sensor based on a piezotransduced single-crystal silicon bulk acoustic resonator (PSBAR). The PSBARs operate at 102 MHz and possess high *Q* values (about 2000), ensuring the stability of the measurement. A corresponding gas sensor array is fabricated by integrating three different self-assembled monolayers (SAMs) modified PSBARs. The limit of detection (LOD) for ethanol vapor is demonstrated to be as low as 25 ppm with a sensitivity of about 1.5 Hz/ppm. Two sets of identification code bars based on the sensitivities and the adsorption energy constants are utilized to successfully discriminate isopropanol (IPA), ethanol, hexane and heptane vapors at low and high gas partial pressures, respectively. The proposed sensor array shows the potential to form a portable electronic nose system for volatile organic compound (VOC) differentiation.

## 1. Introduction

Volatile organic compounds (VOCs) are hazardous materials that have proven to have negative effects on the environment and human health. Concurrently, as sensitive biochemical markers, VOCs are widely used as analytes in the realm of environment protection [1], food testing [2,3,4], early diagnosis [5,6,7,8,9], and so forth. A successful platform for VOC detections is the electronic nose (e-nose) system [10,11] which consists of several sensors modified with different gas-sensitive materials. Numerous gas sensor types have been demonstrated to meet various measurement requirements. Gas sensors based on the metal oxide semiconductor possess a wide range of target gases with satisfactory sensitivity and selectivity, which makes them the most commonly used gas sensors [12]. Nano-size gas sensors, such as carbon nanotubes and graphene, can detect ultra-low concentrations of vapors due to their high surface-area-to-volume ratio [13,14]. Optical gas sensors benefit from the high fidelity of optical signals, making them suitable for remote detections [15]. Microwave gas sensors are emerging as cheap and label-free techniques, and the lack of selectivity can be overcome by combining with highly selective materials [16,17,18,19,20]. Among different types of gas sensors, MEMS piezoelectric gas sensors, such as surface acoustic wave (SAW) resonators [21], Lamb wave resonators (LWR) [22] and film bulk acoustic resonators (FBAR) [23,24,25] have triggered a lot research interest due to their low power consumption, micrometer-scaled sizes, and relatively high sensitivities. Compared with quartz crystal microbalance (QCM), however, they suffer from relatively low *Q* values, which may result in poor limit of detection (LOD), large phase noise, and instability when integrating with oscillating circuits.

As a special acoustic device, the piezotransduced silicon bulk acoustic resonator (PSBAR) not only inherits the characteristics of MEMS acoustic wave devices, but exhibits significant advantages in the aspect of superior *Q* value due to the existence of the single-crystal silicon substrate. Because of their high *Q* factor and relatively low motional resistance, PSBARs have been used as critical elements in high-performance oscillators [26]. Moreover, studies regarding their biochemical sensing capability in liquid environment have been reported [27]. However, the investigations of PSBARs for gas sensing applications are less covered. 

In this work, we designed and fabricated high performance PSBARs operating at the first and third order width-extensional mode (WE mode) with *Q* values up to 12,000 and 2000, respectively. Three kinds of self-assemble monolayers (SAMs), including (3-glycidyloxypropyl) trimethoxysilane (GPTES), trimethoxy (octadecyl) silane (OTES), and (3-bromopropyl) trichlorosilane (BPTS) are functionalized onto the surface of the PSBARs to compose a novel gas sensor array. The comparative detections of the two modes towards low concentration ethanol vapors demonstrate the superior LOD. Finally, the PSBAR array is used to successfully discriminate ethanol, IPA, heptane and hexane at low and high gas partial pressures, showing a great potential as a promising method for VOCs detections. 

## 2. Materials and Methods

### 2.1. Materials

(3-glycidyloxypropyl) trimethoxysilane (GPTES), trimethoxy (octadecyl) silane (OTES) and (3-bromopropyl) trichlorosilane (BPTS) are purchased from Aladdin Industrial Corporation (Shanghai, China) without further purification. VOCs (ethanol, IPA, heptane and hexane) utilized in this work are purchased from Tianjin Real & Lead Chemical Corporation and the purity all reached HPLC.

### 2.2. PSBAR Fabrication

PSBARs are fabricated using SOI wafer by a standard semiconductor processing flow. The device layer of the SOI wafer is 25 μm n-type low-resistivity, single-crystal silicon. To fabricate PSBARs, 0.2 μm molybdenum (Mo), 1 μm c-axis oriented aluminum nitride (AlN) and 0.2 μm molybdenum (Mo) were deposited and patterned as bottom electrodes, piezoelectric layer and top electrodes, respectively. Bottom cavities and the trenches on both sides of a resonator were fabricated by means of the DRIE process. 0.3 μm gold (Au) pads were fabricated using lift-off process. A buried silicon oxide layer was released by a BOE solution to suspend the silicon block in the last step. The length of the resonator was aligned along <110> crystal orientation. The detailed schematic of the PSBAR fabrication is shown in Supplementary Figure S1.

### 2.3. Device Functionalization

To form OTES and BPTS membranes on the surface, PSBARs were rinsed using deionized (DI) water followed by drying in nitrogen. The devices were oxidized in air plasma for 5 min with plasma cleaner (YZD08-2C, SAOT, Beijing, China), and silanization was accomplished by vapor phase deposition of a silylating reagent in a low-pressure heated chamber (YES-LabKote, Yield Engineering Systems, Livermore, CA, USA). The functionalization process of GPTES was the same with OTES and BPTS apart from further reaction with aqueous ethanolamine solutions (20%) for 2 h to form hydroxyl membrane. All the devices were preserved in the nitrogen environment (e.g., glove box) to protect SAMs from oxidation and hydrolysis damage.

### 2.4. Surface Characterization

The characterization of different SAMs were employed by contact angle measurement (JC2000DM, Zhongchen, China). As shown in Supplement Figure S3, the contact angle of the bare silicon substrate is 35.25°. After being functionalized with OTES, GPTES and BPTS, the contact angles increase to 73.17°, 63.04° and 92.56° respectively, which means SAMs were successfully coated. The contact angles of OTES- and BPTS-modified surfaces are larger than GPTES-modified surface due to the higher hydrophobicity of the terminated chemical groups.

### 2.5. VOC Detection Setup

**Figure 1:**
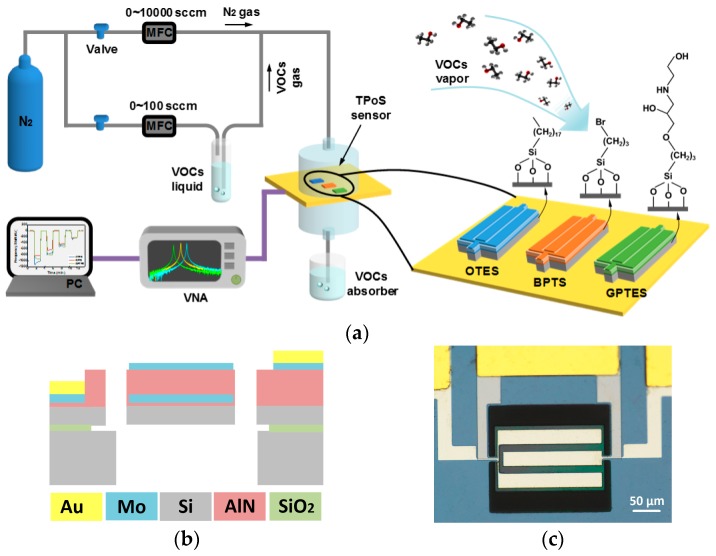
(**a**) Schematic of the gas sensing setup and a piezotransduced silicon bulk acoustic resonator (PSBAR) sensor array; (**b**) Schematic of the PSBAR structure; (**c**) An optical microscope graph of a PSBAR 120 μm in width and 200 μm in length.

The VOCs detection setup consists of a dual-line VOC generation system and a frequency record system, as shown in Figure 1a. In the VOC generation system, an organic solution was added into a bubbler, and pure carrier nitrogen gas was guided into the liquid to generate saturated VOC vapors. Then, VOC vapors with different ratios of partial pressures to saturated vapor pressure (P/P_0_) were realized by diluting the saturated vapor using pure nitrogen in another channel. The real-time flow velocity was monitored by mass flow controllers (MFC, 5850e, Brooks, Hatfield, PA, USA) through a computer program. The VOC vapors were guided to an evaluation board with functionalized PSBARs wire-bonded onto it. The board was epoxied with two plastic cavities in order to confine VOC vapors, as shown in Supplementary Figure S4. A VOC absorber was placed behind the evaluation board to prevent the diffusion of harmful VOCs. In the frequency record system, a vector network analyzer (VNA, E5071C, Agilent, Santa Clara, CA, USA) was connected to the evaluation board. The two-port S-parameter data of each PSBAR were recorded by a program.

### 2.6. Finite Element Analysis Model

Due to the symmetry of the PSBAR structure, a quarter of a 3D model was constructed to reduce the consumption of calculation resources, as shown in Supplementary Figure S2. The piezoelectric transducer, the anisotropic single crystal silicon block, and the centrally located tether were built up. The support tether was clamped with perfect match layer (PML) to simulate the adsorption of acoustic waves by the silicon substrate.

### 2.7. Principal Component Analysis

PCA is a robust pattern recognition tool for classification of multivariate data. It provides an efficient approach to reduce the dimensionality of a data matrix. The first two eigenvalues of the data matrix are calculated as new coordinate bases, which are called the first principal component (PC1) and the second principal component (PC2).

## 3. Results and Discussions

### 3.1. PSBAR Performance Simulations and Device Selections

A PSBAR comprises two parts: a sandwich-form transducer and an attached suspending single-crystal silicon substrate. The transducer consists of a thin-film piezoelectric layer, top and bottom metallic electrodes, as shown in Figure 1b,c. When stimulated by an alternating voltage, the AlN layer produces alternating stress due to its piezoelectric effect, which leads to mechanical waves propagating in the silicon substrate. Owing to the finite size, the mechanical waves form standing waves at special stimulating frequencies, which results in resonant peaks in the frequency spectrum. When gas molecules are absorbed on the device surface, the resonant peaks shift downwards due to the mass loading effect. The relation between the absorbed mass and the frequency shifts can be described by the Sauerbrey equation [28] as following:

(1) $$\Delta f=\frac{-2f_{0}^{2}}{A\sqrt{\mu_{eff}\rho }}\Delta m=\frac{-2f_{0}^{2}}{A\rho v_{a,eff}}\Delta m,$$

where Δ*f* denotes the measured frequency shift; *f*_0_ is the intrinsic resonant frequency of each mode; Δ*m* is the mass change; *A* is the effective sensing area; *μ_eff_* is the effective Young’s modulus of the resonator along the direction of acoustic wave propagation; *ρ* is the density of the material. Alternatively, the equation can be written as a function of *v_a,eff_* (effective acoustic phase velocity). Therefore, by detecting the frequency shifts, the amount of adsorbed gas can be extracted. 

A vital parameter for a mass sensor is the LOD, which is closely related to the minimum detectable resonant frequency change (Δ*f*_min_). Δ*f*_min_ is influenced by multiple factors, such as resonator *Q* values, sensing membranes, ambient environment conditions, and the system noise of the measurement equipment. In practice, the Δ*f*_min_ can be calculated in terms of *Q* and minimum detectable phase shift of impedance ($\Delta \phi_{\min}$) as follows:

(2) $$\Delta f_{\min}=\frac{\Delta \phi_{\min}}{2Q}.$$

Hence, a high *Q* factor can reduce the LOD of the sensor. Moreover, when integrating sensors with measurement circuits, a high *Q* factor can reduce the noise and enhance the stability. Therefore, to get better sensing performance, a high *Q* value of the PSBAR is desired.

**Figure 2:**
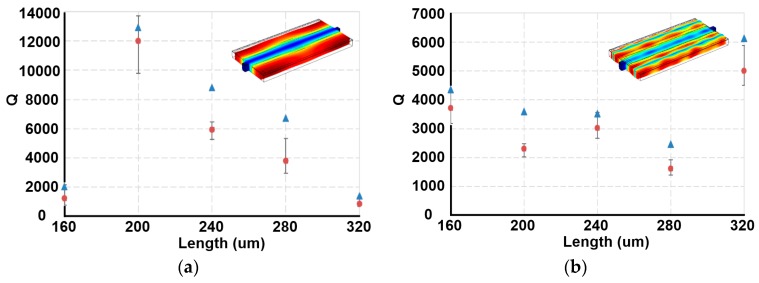
Simulated and measured *Q* values of PSBARs with different length. The width is 120 μm. *Q* values of (**a**) first order width-extensional mode (WE mode) and (**b**) third order WE mode. The triangle markers represent simulation results. The insets are the displacements of PSBARs at the first and third order WE modes.

In order to determine the optimum size of PSBAR sensors, a finite element analysis model was built up. The width of the PSBAR model is 120 μm. By sweeping the length of the PSBAR model, a set of *Q* factors of the first and third order WE mode can be calculated, as shown in Figure 2. 

It shows that the *Q* factor of the first order WE mode reaches maximum (12,927) when the length is 200 μm. Although 320 μm length PSBARs possess the highest *Q* (6129) of the third order WE mode, the *Q* value of its first order WE mode is rather low. Therefore, the 200 μm length PSBAR is preferable. 

To verify the simulation results, PSBARs with different length were fabricated and their statistical *Q* values are plotted in the same figure. The variation trend of the statistical *Q* values is in accordance with simulation results except that they are slightly smaller than the theoretical calculations, which is due to the loss of the materials, lattice defect, and electrode resistivity in practice. 

Three PSBARs (200 μm in length) with similar performances were selected to compose a gas sensor array. Their performances are shown in Supplementary Figure S5. The operating frequencies of the first order WE mode are about 35.6 MHz, and the frequencies of the third order WE mode are around 102 MHz. The *Q* values of the first order WE mode are larger than 12,000, and the *Q* values of the third order WE mode are around 2000. The high *Q* factors ensure their outstanding detection capability when exposed to VOCs. 

### 3.2. Comparative Detections of Low-Concentration Ethanol Vapor

**Figure 3:**
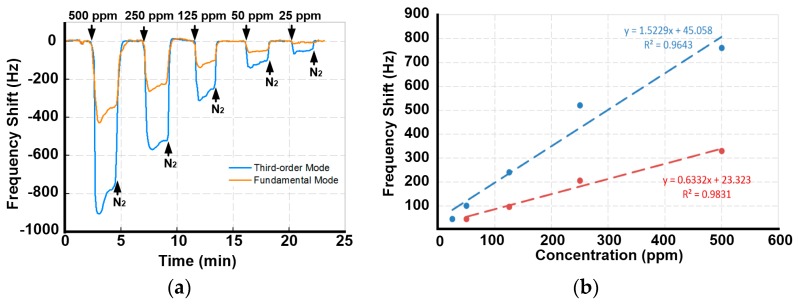
(**a**) Real-time responses of the first and third order WE modes of the trimethoxy (octadecyl) silane (OTES)-modified PSBAR to low-concentration ethanol vapors; (**b**) Sensitivities of the two sensing modes. R^2^ is the correlation coefficient.

In order to compare the sensing capability of the first and third order WE modes, a PSBAR modified with BPTS was used to detect low concentration ethanol vapor. A 2000 ppm standard ethanol gas was prepared and connected to the VOC channel. By diluting the standard ethanol gas with pure nitrogen, 500 ppm, 250 ppm, 125 ppm, 50 ppm and 25 ppm ethanol gases were generated and detected sequentially with the PSBAR sensor. The real-time sensing results are shown in Figure 3a.

The results show that, when nitrogen is guided to the sensor, the resonant frequencies reach stable baselines. When the sensor is exposed to ethanol vapors, the resonant frequencies decreases immediately, indicating a quick adsorption of ethanol molecules. After flushing with nitrogen, the resonant frequencies recover rapidly, indicating the full desorption of ethanol molecules. The fast adsorption and desorption processes demonstrate the good repeatability and stability of PSBAR sensors.

The frequency shifts of the third order WE mode are always larger than that of the first order mode because of the higher working frequency, which is in accordance with Equation (1). When the sensor is exposed to 25 ppm ethanol gas, the resonant frequency of the third order WE mode still decreases by 46 Hz, while the response of the first order WE mode is hardly to be discerned. This is mainly due to the fact that, although the first order WE mode possesses higher *Q* value, the responses are limited by the resolution of the VNA. The third order WE mode, however, can still be detected due to the higher sensitivity. To further investigate the sensitivity of each mode, the frequency shifts versus concentrations are depicted in Figure 3b. It shows that the third order WE mode has a sensitivity about 1.52 Hz/ppm, which is almost three times higher than that of the first order WE mode. Therefore, the third order WE mode is used as sensing mode in the following VOC detections. 

### 3.3. Discriminations for Different VOCs at Low Gas Partial Pressures

**Figure 4:**
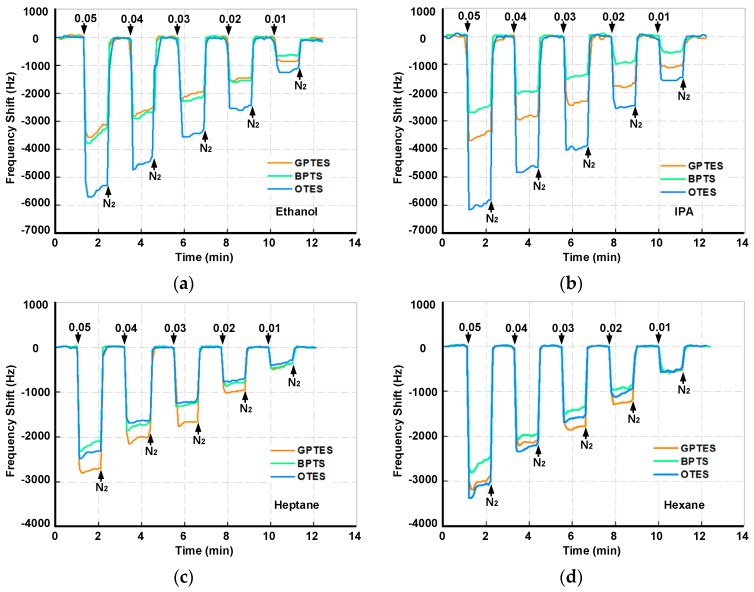
Real-time responses of the PSBAR gas sensor array to (**a**) ethanol, (**b**) IPA, (**c**) heptane and (**d**) hexane.

To realize the VOC differentiations, the three selected PSBARs were modified with OTES, GPTES and BPTS, respectively, to form a gas sensor array. The sensor array was exposed to four kinds of VOCs (ethanol, IPA, heptane, hexane) with gas partial pressures varying from 0.05 to 0.01. Figure 4 shows real-time frequency responses of the PSBAR sensor array. It is intuitive to note that different SAM-modified PSBARs have different responses towards each VOC, which mainly results from the discrepancies of the amphipathicity between VOCs molecules and the three SAMs. For polar VOCs (ethanol and IPA), the OTES-modified PSBAR shows maximum responses at about 5.3 kHz and 5.8 kHz, respectively, under 0.05 gas partial pressure, while the maximum frequency shifts of OTES-modified PSBAR to nonpolar vapors (hexane and heptane) are only 2.4 kHz and 3.1 kHz, respectively, which means OTES has higher adsorption volume to polar vapors at low gas partial pressures.

**Figure 5:**
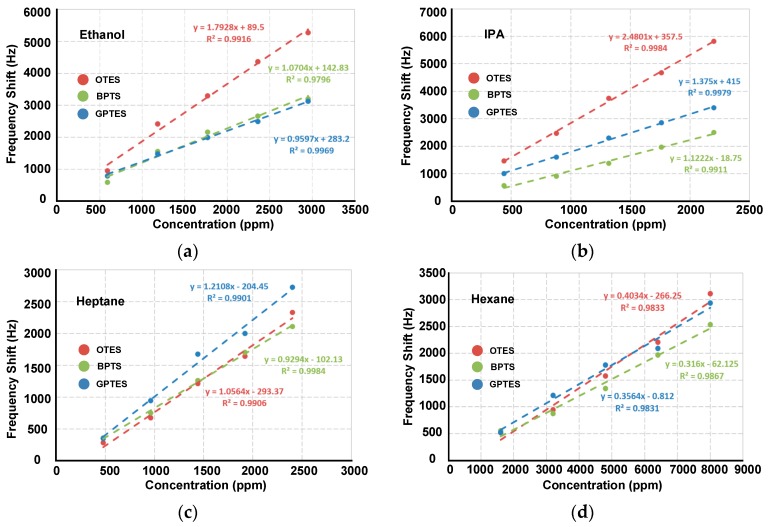
Linear fitting of sensitivities of three self-assembled monolayers (SAM)-modified PSBARs to (**a**) ethanol, (**b**) IPA, (**c**) heptane and (**d**) hexane. R^2^ is the correlation coefficient.

To calculate the sensitivity of each PSBAR and generate the code bars for VOC differentiation, the concentrations of VOC vapors in parts per million (ppm) were calculated by the following equation:

(3) $$C(ppm)=10^{6}\times (P_{s}f/P(f+F)),$$

where *f* and *F* are the flow rates (in sccm) of saturated VOCs and dilution nitrogen, respectively; *P* is the standard atmospheric pressure (760 mmHg). *P_S_* is the saturated partial vapor pressure obtained using the Antoine equation [29]:

(4) $$\log P_{s}=A-\frac{B}{t+C},$$

where *t* (°C) is the measurement temperature. *A*, *B*, *C* are empirical coefficient related to the detected vapors. By referring to the chemical handbook, the *Ps* of ethanol, IPA, heptane and hexane are calculated to be 36.48, 33.44, 36.48, and 121.6 mmHg, respectively. Therefore, the concentrations under 0.05 gas partial pressure of ethanol, IPA, heptane, and hexane are 2950, 2200, 2400, and 8000 ppm, respectively. The sensitivities of PSBARs to four VOCs can be depicted as Figure 5. 

**Figure 6:**
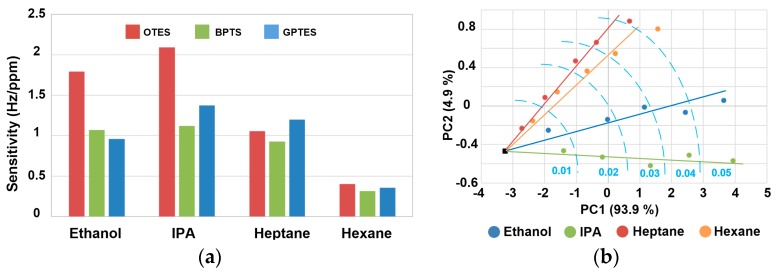
(**a**) Identification code bars for detected volatile organic compounds (VOCs); (**b**) Score plots of detected VOCs calculated by Principal Component Analysis (PCA) method.

Least square method is used to linearly fit the data. Figure 5 shows that the sensitivities of three SAM-modified PSBARs to each VOC are distinctive from each other, which represents three non-redundant variables. As a result, the sensitivities can be used to form identification code bars for VOC differentiations. The code bars for four VOCs are shown in Figure 6a. 

It clearly shows that the code bars for four VOCs have obvious dissimilarity. For polar vapors (ethanol and IPA), the sensitivity of OTES-modified device is the highest, which is in agreement with the real-time sensing results. Furthermore, ethanol and IPA can be differentiated by comparing the magnitude of sensitivities of BPTS- and GPTES-modified sensors: if the sensitivity of GPTES-modified PSBAR is larger, the analyte is IPA, otherwise, it is ethanol. Although the code bars of hexane and IPA share the similar pattern, the differences between sensitivities of OTES- and BPTS-modified sensors can still be used to realize the differentiation. For heptane, the maximum response occurs at GPTES-modified sensor. Hence, it is the most recognizable vapor among detected VOCs. 

In order to quantitatively assess the discriminations towards different VOCs, Principal Component Analysis (PCA) algorithm was applied to process the data. A 21 × 3 data matrix is built up as shown in Supplementary Table S1. The row variables are four VOC species under five gas partial pressures, and the column variables are the three SAMs. Zeros are added to the last row in order to represent the blank responses. The transformation results are plotted in Figure 6b. 

The black point in the figure is the blank responses. The results show that the four different vapors form individual response directions, which means the PSBAR sensor array successfully differentiates between the four VOCs. Besides, the data points of each VOC arrange in a linear format from 0.01 to 0.05 gas partial pressures and radiate from the blank point, illustrating the superior linearity of the PSBAR sensor array. In short, the code bars and PCA results prove the preferable discrimination capability and linearity of PSBAR sensor array for VOC sensing at low gas partial pressures.

### 3.4. Differentiations for Different VOCs at High Gas Partial Pressures

As demonstrated above, the sensitivity-based code bars can successfully differentiate between VOCs within a narrow range of gas partial pressures. When differentiating between VOCs within a large range of gas partial pressures, however, such code bars are ineffective due to the nonlinearity of the PSBAR responses. Thus, to differentiate between VOCs at high gas partial pressures, concentration-independent code bars are desired. Here, we use the fitting results from the adsorption isotherms to generate the unique concentration-independent code bars for detected VOCs.

**Figure 7:**
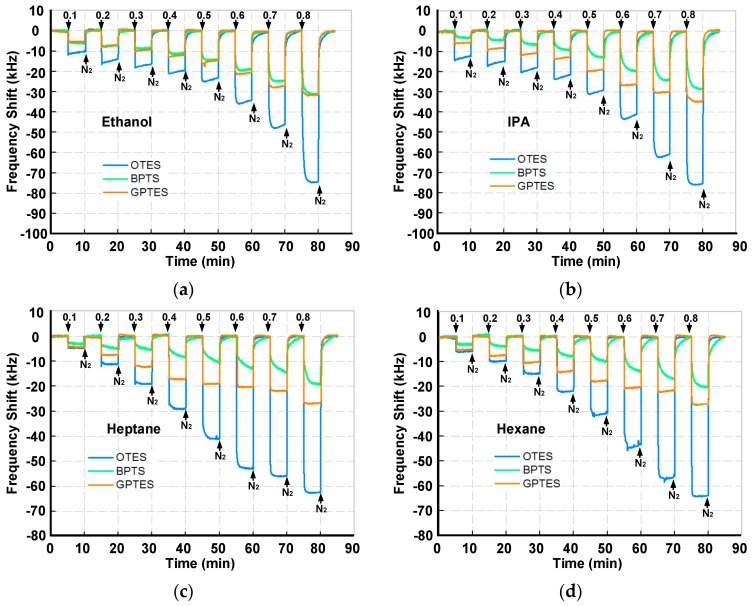
Real-time responses of the PSBAR array for detections of (**a**) ethanol, (**b**) IPA, (**c**) heptane and (**d**) hexane with gas partial pressures varying from 0.1 to 0.8.

Figure 7 shows the real-time responses of the PSBAR array to four VOCs (ethanol, IPA, heptane and hexane). It clearly shows that the adsorption and desorption of VOCs on the SAM-modified PSBAR array are reversible processes, even at high gas partial pressures. Moreover, with the increase of the gas partial pressures, the amount of the VOCs’ adsorptions grows. Among the three SAM-modified sensors, OTES-modified PSBAR possesses the highest magnitude of responses when gas partial pressures are greater than 0.2, which may result from the longer chain length of OTES molecules. This effect is not obvious at low gas partial pressure due to the relatively low concentrations. With the increase of gas partial pressures, however, chain length becomes a dominant factor, which, together with the amphipathicity between VOC molecules and modified SAMs, ultimately contributes to the disparate responses of the three sensors. Moreover, it seems that when gas partial pressures are low, the adsorption responses do not follow exponential patterns. This might be due to the flow fluctuations of the gas sensing setup. The response time and recovery time were defined as the time required to change the frequency after exposure to VOCs or nitrogen in a specific range of 90%, as illustrated in Figure S7a. At 0.8 gas partial pressure, GPTES-modified PSBAR exhibits the shortest response time, while BPTS-modified PSBAR owns the longest response time. All the response and recovery times for VOCs at 0.8 gas partial pressures are given in Table S2. Additionally, during the measurement, the *Q* values of the PSBARs have relatively small fluctuations as shown in Figure S8, which ensures the high performance when integrating with oscillator circuits.

**Figure 8:**
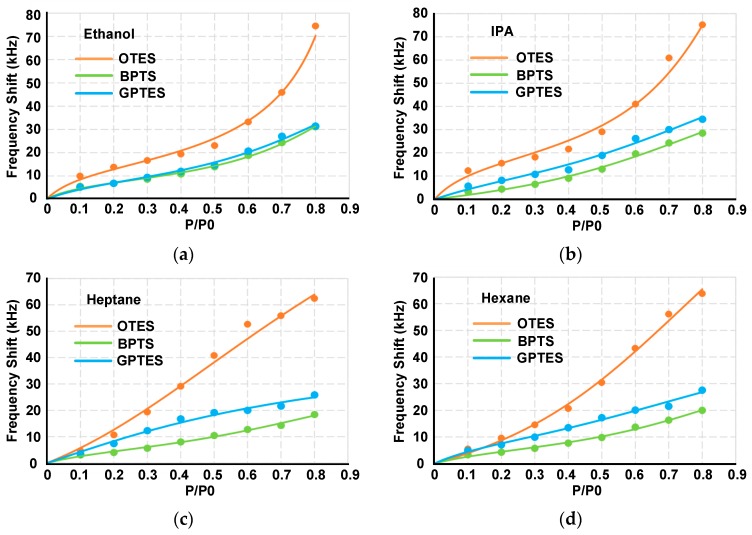
Adsorption isotherms of VOCs: (**a**) ethanol, (**b**) IPA, (**c**) heptane and (**d**) hexane.

The adsorption isotherms of each VOC on different sensors can be obtained according to the frequency shifts at different partial pressures, as shown in Figure 8. It shows that the adsorptions of different VOCs fit different adsorption types according to their polarities, which is particularly obvious on the OTES-modified PSBAR. Brunauer-Emmett-Teller (BET) formula with finite adsorbed layers is used to fit the adsorption isotherms, which is the typical model of multilayer gas physical adsorption:

(5) $$\Delta f\propto v=\frac{v_{m}cx}{1-x}\cdot \frac{1-(n+1)x^{n}+nx^{n+1}}{1+(c-1)x-cx^{n+1}},$$

where *v* is the total gas volume adsorbed; Δ*f* is the frequency shift of each mode, which is linearly proportional to *v*; *v_m_* is the monomolecular layer adsorption capacity; *x* is the gas partial pressure; *c* is the adsorption energy constant; and *n* is the maximum number of layers that can be reached. In the BET model, the constant *c* describes the adsorption energy difference between the first layer and the subsequent layers, which is approximately given by

(6) $$c\approx e^{(q_{1}-q_{L})/RT},$$

where *q*_1_ is the heat of adsorption in the first layer on the surface, which represents the interaction force between the SAMs and VOC molecules. While the *q_L_* is the condensation heat of subsequent layers, which represents the interaction forces between the VOC molecules. The fitting curves of four VOCs are shown in Figure 8.

**Figure 9:**
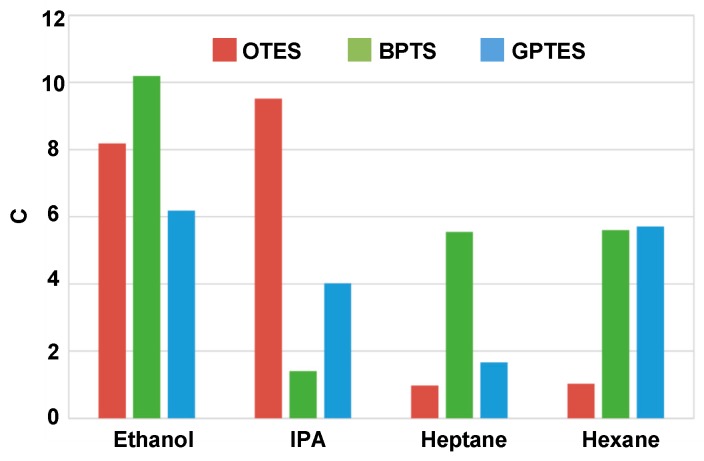
Concentration-independent code bars.

After extracting the *c* values of each isotherms, concentration-independent code bars for four VOCs can be depicted as Figure 9. It shows that when detected by OTES-modified PSBAR, *c* values of polar VOCs (ethanol and IPA) are larger than 1, suggesting that the *q*_1_ is much greater than the *q_L._* For nonpolar VOCs (heptane and hexane), *q*_1_ is closed to *q_L_* making c values approximate 1. The difference is likely due to the fact that interactions between polar molecules and OTES monolayer are larger than that between nonpolar molecules and OTES monolayer. It results that the adsorbed gas molecules increased quickly at low gas partial pressure (typically below 0.1) in adsorption isotherms of polar VOCs, as shown in Figure 8. The concentration-independent code bars for four VOCs are distinctive, which means by simply diluting an unknown VOC target, the absorption isotherms can be obtained and the code bars based on the *c* can be constructed to realize VOC differentiations.

## 4. Conclusions

In this work, high-*Q* PSBARs modified with SAMs are applied as high-performance gas sensors. The influence of length-width ratios on *Q* values is discussed to obtain the optimum size for the PSBAR sensor. The detection of 25 ppm ethanol vapor is realized by the third order WE mode of an OTES-modified PSBAR. A gas sensor array consists of three PSBARs functionalized with three SAMs (OTES, BPTS and GPTES) has been fabricated. By means of extracting the different sensitivities and adsorption energy constant of PSBARs towards different VOCs at low and high gas partial pressures, unique identification code bars for VOCs discriminations can be obtained. Four VOCs (ethanol, IPA, hexane and heptane) have been successfully differentiated, demonstrating SAM-modified PSBAR sensors as promising candidates in VOC detections. 

## Supplementary Materials

The following are available online at http://www.mdpi.com/1424-8220/17/7/1507/s1, Figure S1: schematic of the PSBAR fabrication process flow, Figure S2: finite element model to simulate the *Q* values of different size PSBARs, Figure S3: contact angles of four kinds of interfaces, Figure S4: assembled PSBAR evaluation board, Figure S5: electrical performances of the first and third order WE mode of the three selected PSBARs used in the e-nose system, Figure S6: SEM picture of a side wall of a PSBAR, , Figure S7: Adsorption and desorption response time for (a) ethanol, (b) IPA, (c) heptane and (d) hexane at 0.8 gas partial pressure, Figure S8: *Q* variations when detecting IPA at gas partial pressures from 0.1 to 0.5, Table S1: frequency shifts matrix for PCA transformation, Table S2: Adsorption and desorption response time at 0.8 gas partial pressure.
