# Supplementary material for: Novel Gas Sensor Arrays Based on High-Q SAM-Modified Piezotransduced Single-Crystal Silicon Bulk Acoustic Resonators
Source: Sensors (Basel). 2017 Jun 26;17(7):1507. doi: 10.3390/s17071507 (PMC5539522; doi:10.3390/s17071507)
Supplement: Supplementary file 1 [file sensors-17-01507-s001.pdf]

Novel Gas Sensors and E-nose System Based on High-Q SAM-Modified Piezotransduced Single-Crystal Silicon Bulk Acoustic Resonators

Yuan Zhao †, Qingrui Yang †, Ye Chang, Wei Pang, Hao Zhang, Xuexin Duan *

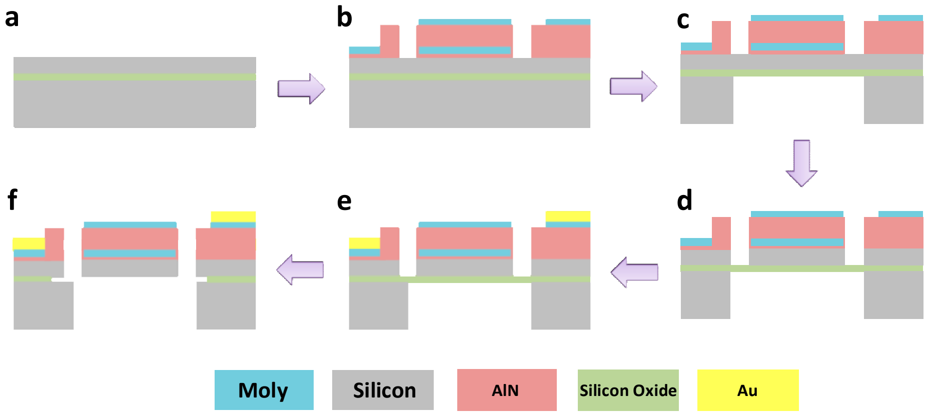

Figure S1. Schematic of the PSBAR fabrication process flow: (**a**) SOI wafer is prepared; (**b**) Bottom electrodes (Mo), piezoelectric layer (AlN) and top electrodes (Mo) are deposited and patterned sequentially; (**c**) handling silicon is removed using DRIE process; (**d**) device silicon layer is etched through DRIE process; (**e**) gold pads are fabricated by means of lift-off process; (**f**) buried oxide layer (SiO_2) is released by BOE solution.

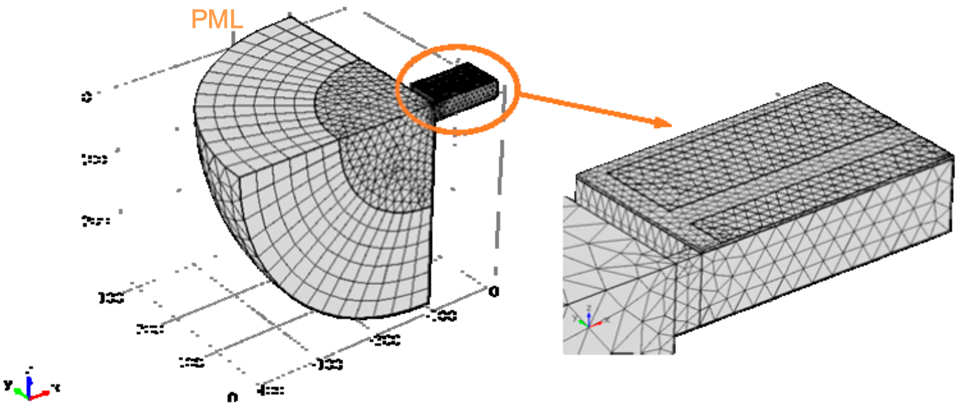

Figure S2. Finite element model to simulate the Q values of different size PSBARs. Only quarter of the device model is built up owning to their asymetrical structure to save calculation resources. A quart of sphere perfect match layer (PML) is attached to the tether to simulate the adsorption of acousitc waves by the silicon substrate. The thickness of electrodes (Mo), piezoelectric layers (AlN), silicon substrate set as 0.2 μm, 1 μm and 25 μm respectively. The width of PSBAR fixed as 120 μm. The center lines of two top electrodes are 40 μm apart. The width of a complete electrode is 30 μm. The size of a complete tether is 10 μm long and 10 μm wide. The crystal orientation of silicon substrate is set as [110]. Two-port S parameter is simulated to further calculate Q value by means of 3dB bandwidth method.

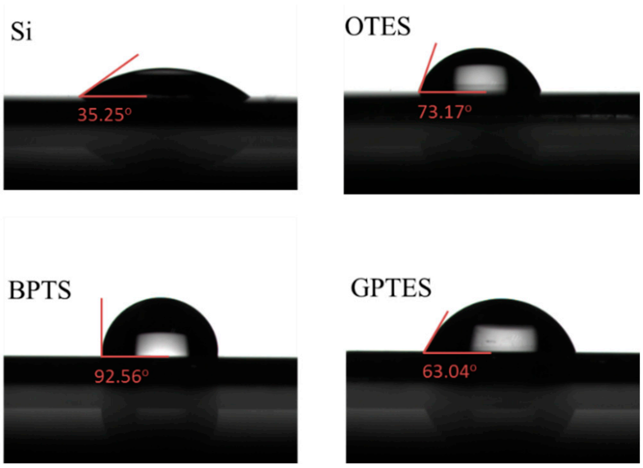

Figure S3. Contact angles of four kinds of interfaces. The hydrophobicity of the interfaces increase after the functionalization indicating the successful modifications of SAMs.

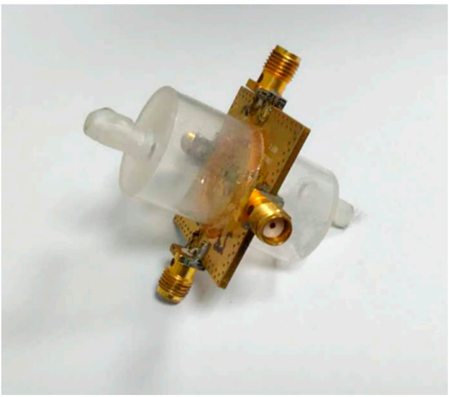

Figure S4. Assembled PSBAR evaluation board. The PSBAR sensing array is wire-bonded onto the evaluation board to connect vector network analyzer through SMA connectors. Plastic chambers are used to make the VOCs thoroughly contact the sensors.

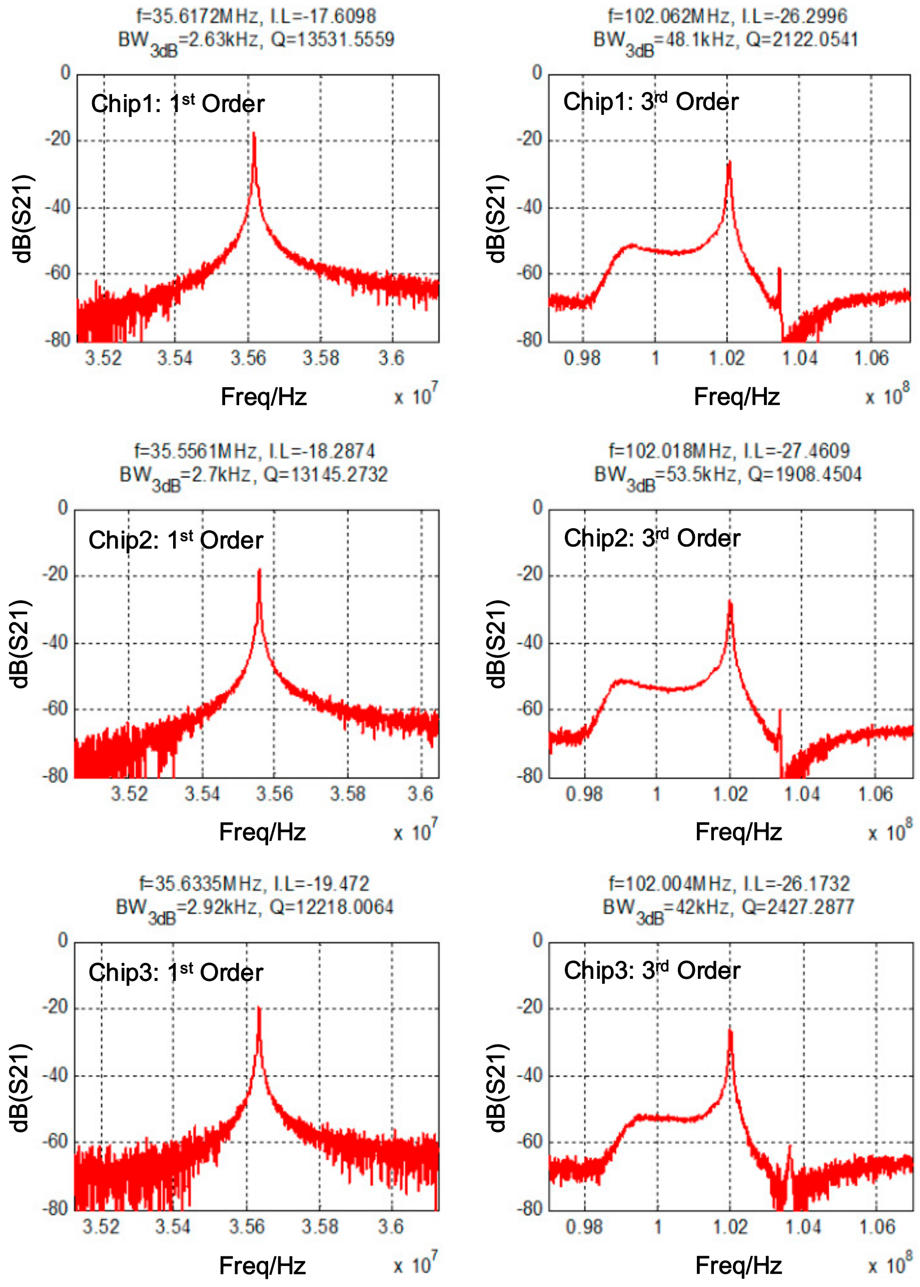

Figure S5. Electrical performances of the first and third order WE mode of the three selected PSBARs used in the e-nose system.

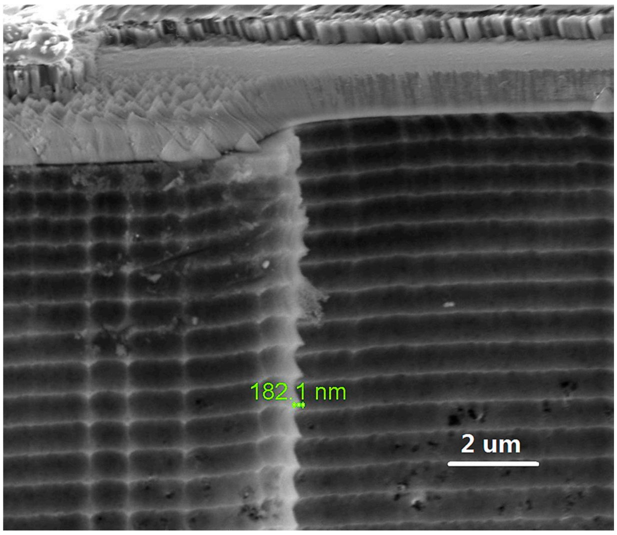

Figure S6. SEM picture of a side wall of a PSBAR. The absorption area expands due to the ripple surface formed by DIRE process.

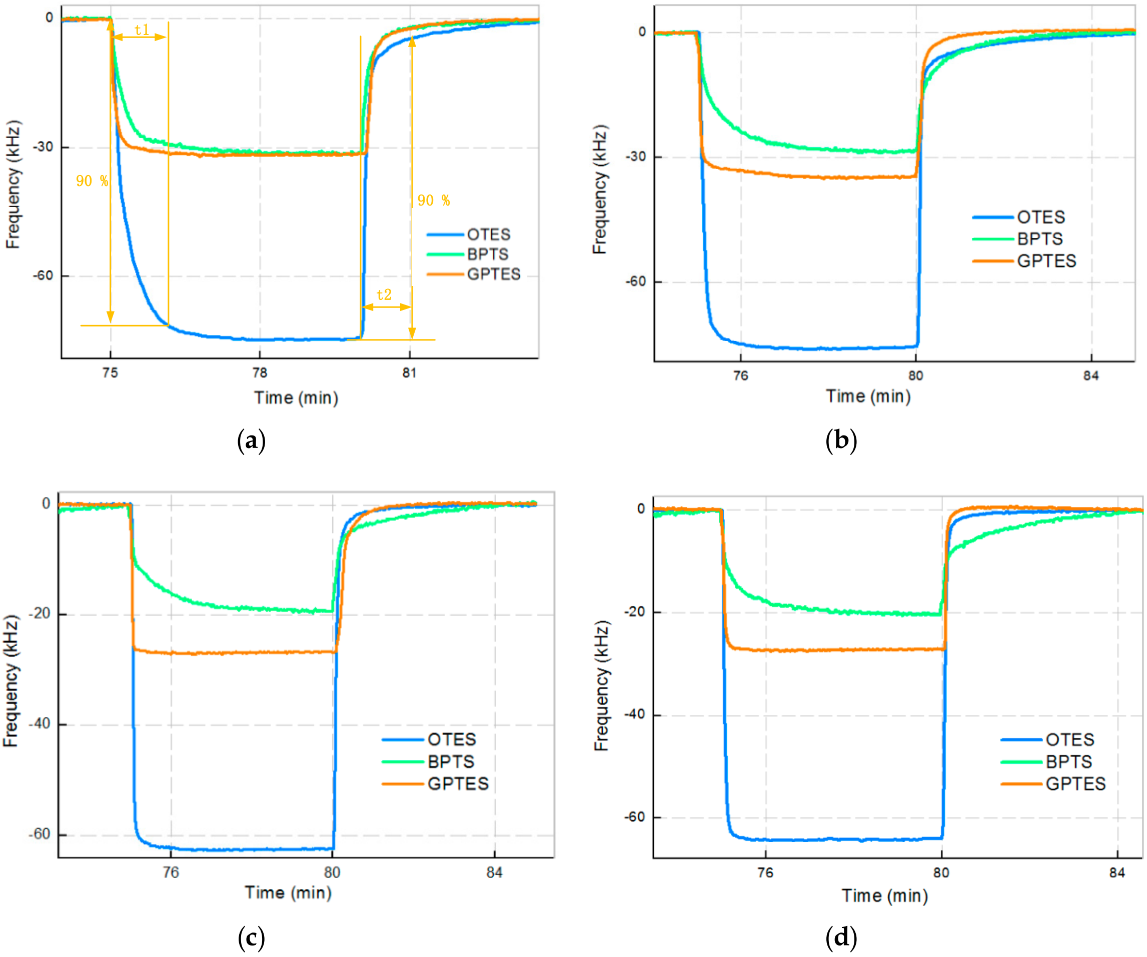

Figure S7. Adsorption and desorption responses for (**a**) ethanol, (**b**) IPA, (**c**) heptane and (**d**) hexane at 0.8 gas partial pressure. The adsorption response time is defined as t_1 and the desorption response time is defined as t_2.

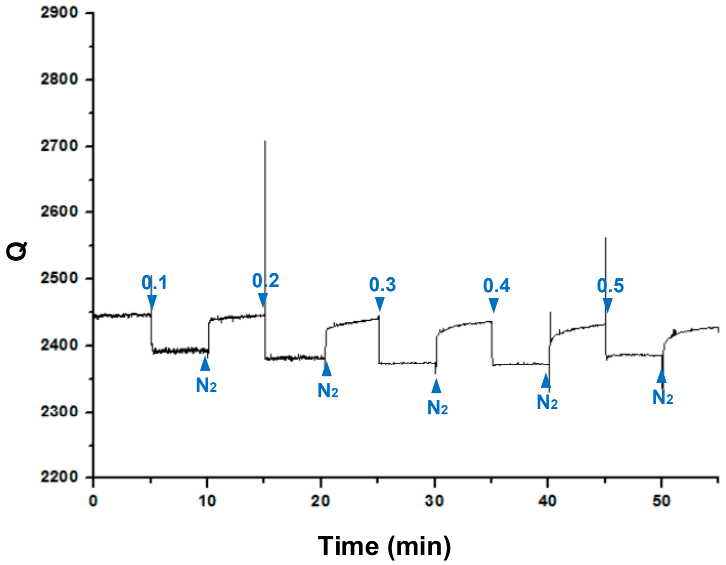

Figure S8. Q variations when detecting IPA at gas partial pressures from 0.1 to 0.5.

Table S1. Frequency shifts matrix for PCA transformation (kHz).

	P/P_0	OTES	BPTS	GPTES
**ethanol**	0.01	0.960	0.594	0.800
	0.02	2.419	1.565	1.486
	0.03	3.294	2.161	2.000
	0.04	4.366	2.666	2.500
	0.05	5.275	3.201	3.124
**IPA**	0.01	1.463	0.563	1.000
	0.02	2.469	0.906	1.600
	0.03	3.738	1.375	2.300
	0.04	4.669	1.969	2.850
	0.05	5.819	2.500	3.400
**Heptane**	0.01	0.281	0.356	0.350
	0.02	0.674	0.750	0.943
	0.03	1.211	1.261	1.674
	0.04	1.640	1.704	2.002
	0.05	2.334	2.110	2.727
**Hexane**	0.01	0.516	0.553	0.525
	0.02	0.945	0.875	1.215
	0.03	1.573	1.344	1.780
	0.04	2.204	1.969	2.091
	0.05	3.114	2.534	2.938
**Blank**		0	0	0

Table S2. Adsorption and desorption response time at 0.8 gas partial pressure

	OTES		BPTS		GPTES	
	t_1	t_2	t_1	t_2	t_1	t_2
**ethanol**	48s	37s	49s	28s	16s	39s
**IPA**	17s	21s	87s	84s	15s	20s
**Heptane**	6s	12s	84s	108s	4s	36s
**Hexane**	8s	8s	60s	144s	7s	9s

t_1: response time, t_2: recovery time
